# Rapid Detection of Adulterants in Whey Protein Supplement by Raman Spectroscopy Combined with Multivariate Analysis

**DOI:** 10.3390/molecules24101889

**Published:** 2019-05-16

**Authors:** Xianzhi Jiao, Yaoyong Meng, Kangkang Wang, Wei Huang, Nan Li, Timon Cheng-Yi Liu

**Affiliations:** 1 MOE Key Laboratory of Laser Life Science & Laboratory of Photonic Chinese Medicine, College of Biophotonics, South China Normal University, Guangdong 510631, China; 13503040644@163.com (X.J.); 15625072392@163.com (K.W.); huangwei2267@163.com (W.H.); linand66@163.com (N.L.); 2 Laboratory of Laser Sports Medicine, South China Normal University, Guangdong 510631, China; liutcy@scnu.edu.cn

**Keywords:** whey protein supplement, Raman spectroscopy, chemometrics, rapid adulteration analysis, creatine, l-glutamine, taurine

## Abstract

The growing demand for whey protein supplements has made them the target of adulteration with cheap substances. Therefore, Raman spectroscopy in tandem with chemometrics was proposed to simultaneously detect and quantify three common adulterants (creatine, l-glutamine and taurine) in whey protein concentrate (WPC) powder. Soft independent modeling class analogy (SIMCA) and partial least squares discriminant analysis (PLS-DA) models were built based on two spectral regions (400–1800 cm^−1^ and 500–1100 cm^−1^) to classify different types of adulterated samples. The most effective was the SIMCA model in 500–1100 cm^−1^ with an accuracy of 96.9% and an error rate of 5%. Partial least squares regression (PLSR) models for each adulterant were developed using two different Raman spectral ranges (400–1800 cm^−1^ and selected specific region) and data pretreatment methods. The determination coefficients (R^2^) of all models were higher than 0.96. PLSR models based on typical Raman regions (500–1100 cm^−1^ for creatine and taurine, the combination of range 800–1000 cm^−1^ and 1300–1500 cm^−1^ for glutamine) were superior to models in the full spectrum. The lowest root mean squared error of prediction (RMSEP) was 0.21%, 0.33%, 0.42% for creatine, taurine and glutamine, and the corresponding limit of detection (LOD) values for them were 0.53%, 0.71% and 1.13%, respectively. This proves that Raman spectroscopy with the help of multivariate approaches is a powerful method to detect adulterants in WPC.

## 1. Introduction

The quality and safety of products have always been the focus of attention in the food field. Whether the ingredients of the food are consistent with the label and whether harmful substances are added to the food is just as important for consumers, distributors and producers. Food fraud can cause serious economic losses and even threaten the health of consumers. In 2007, wheat gluten and rice protein concentrate containing large amounts of melamine and cyanuric acid were used in pet foods, causing a number of pet death [1]. The melamine incident in China sickened hundreds of thousands of infants [2]. Many of the finished foods were recalled in the past few years due to intentional adulteration [3]. In order to protect the health and economy of their citizens, some countries have adopted trade restrictions to prevent similar tragedies. Therefore, detecting the quality of food to avoid food adulteration is very significant for both the government and consumers.

Recently, increasing consumer awareness regarding health and fitness has been leading to a remarkable increase in the consumption of sports supplements. Whey protein sports supplements are favored by athletes and fitness enthusiasts for its ability to replenish proteins needed for human exercise and contribute to improvement in athletic performance [4]. Whey protein concentrate (WPC) powder, which contains 50–85% of protein on a dry basis [5], is one of the most common sports supplements.

Protein content is one of the main quality index of protein-based products [4,6] and an important message to communicate with consumers. It is commonly measured by the traditional Kjeldahl and Dumas methods, which determine the protein content of a sample by directly measuring the total nitrogen content in it [7,8]. These methods do not have the ability to distinguish the protein nitrogen from the non-protein nitrogen [9]. Therefore, the defects of these measurement methods provide the possibility for potential adulteration. Some unscrupulous producers have been motivated by commercial interests to maliciously add cheap soy protein powder or nitrogen rich substances (urea, melamine) to whey protein powder in order to cover up the adulteration and obtain fake high protein test results [10,11,12]. At present, adding inexpensive amino acids and amino acid derivatives to protein powder products to modify protein content has become a common adulteration method. Creatine, taurine and L-glutamine are common amino acids and amino acid derivative adulterated in whey protein-based sports supplements because they are not only cheaper than whey protein and rich in nitrogen, but they are also presented as improving athletes’ athletic ability and facilitating recovery after exercise.

Committed to detecting and quantifying a certain individual nitrogen-based adulterant within the complex mixture of dairy products was already accomplished. Methods such as fluorescence spectra [13,14], microfluidic immunosensor [15], ultraviolet visible spectra [16,17], molecularly imprinted electrochemical sensors [18], high-performance liquid chromatography/compact mass spectrometry [10,19] have been widely used to characterize the adulterants in dairy food. However, these methods have many disadvantages, such as time consumption, the need for well-trained manpower and the high cost of experiments. Therefore, faster, low-cost and more accurate methods for detecting adulterants are urgently needed. Raman spectroscopy is a powerful analytical tool for rapid, nondestructive detection of solid or liquid samples, and has been successfully used to detect adulteration in a variety of complex foods [20,21]. In previous studies, Jianwei Qin proved that Raman chemical imaging with the help of a proper mixture of analysis algorithms could effectively detect multiple adulterated substances in food powder [21]. Raman spectroscopy was also successfully applied to the rapid quantitative detection of adulterated urea in liquid milk and resulted an accuracy of over 90% [12]. Based on the above, the detection of adulterants in whey protein powder by Raman spectroscopy has a great possibility of success.

The combination of Raman spectroscopy with multivariate analyses, for example, principal component analysis (PCA), partial least squares discriminant analysis (PLS-DA), soft independent modeling class analogy (SIMCA) and partial least squares regression (PLSR) can yield strong analytical results and have been widely used in various fields. Principal component analysis (PCA) is an unsupervised analytical method that can reduce the dimensionality of data into irrelevant principal components (PCs) and visualize the relationships between samples [22]. PLS-DA and SIMCA are supervised statistical methods, which are widely used in pattern recognition. The difference between the two methods is that PLS-DA relies on the partial least squares (PLS) regression algorithm, which establishes a PLSR model for all classes from the given data set. While SIMCA is based on PCA, which establishes PCA models for each class in the suggested data set [23]. The output of the PLS-DA model results consists of three steps. The first step, also known as the calibration step, is to optimize the parameters by performing the algorithm. The second step of the algorithm is called the validation step and it uses the regressors obtained in the calibration step to predict the class of samples that do not participate in the calibration step. The final step of the algorithm is to compare the predicted class of the sample with its actual class, and calculate several relevant error parameters. Output steps for the results of the SIMCA model are similar. PLS-DA or SIMCA calibration data sets contain an X matrix of size I*J (I refers to the number of spectra, J is the number of spectral variables), which were established using Raman spectra of the tested samples, and a Y matrix containing predefined classification information of each calibration sample.

Therefore, the present work aimed to explore the applicability of Raman spectroscopy coupled with chemometrics to detect and quantify multiple adulterants in WPC. The two main objectives were to develop the classification model (PLS-DA and SIMCA) to distinguish different types of adulteration and construct a PLSR model using Raman data to quickly determine the adulteration level in WPC.

## 2. Results and Discussion

### 2.1. Raman Spectra

**Figure 1:**
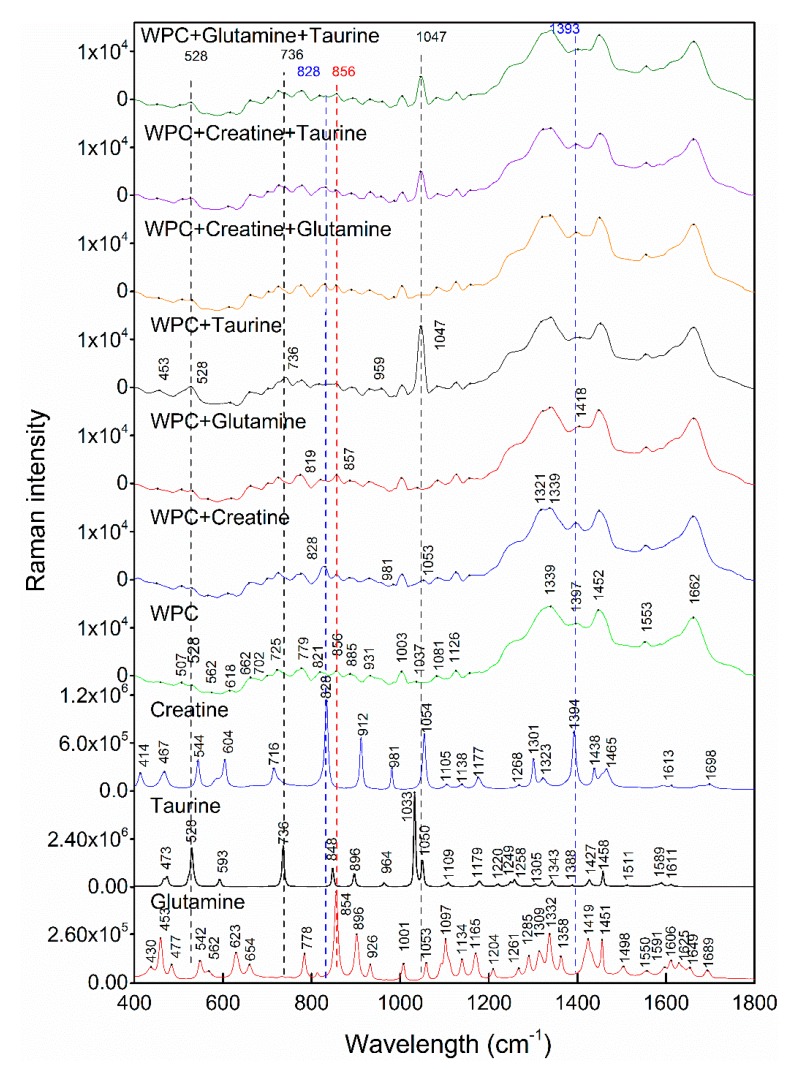
Pretreated Raman spectra of mixture samples with the highest adulteration levels and individual adulterants.

Figure 1 represents the Raman spectra collected from pure WPC, creatine, taurine and glutamine, as well as the processed (baseline correction and smooth) Raman spectra of mixtures with the highest adulteration levels, respectively. There are numerous Raman scattering peaks in the range of 400–1800 cm^−1^ for each substance. The absorption peaks on the spectra were marked with black dots and the main peaks were specially highlighted by a colored vertical line. In this paper, W (WPC), WG (WPC + glutamine), WC (WPC + creatine), WT (WPC + taurine), WCG (WPC + creatine + glutamine), WCT (WPC + creatine + taurine) and WGT (WPC + glutamine + taurine) were used as symbols to indicate real information of each sample. W, C, G and T refer to whey protein concentrate, creatine, glutamine and taurine, respectively. Typical peaks of taurine appeared at 528 cm^−1^, 736 cm^−1^ and 1033 cm^−1^, which was in accordance with the result of Moreira’s study. They correspond to the δ_s_ (SO_3_), *ν* (CS) and *ν* (CN), respectively [24]. Peaks at 528 cm^−1^ and 736 cm^−1^ were visible not only in samples containing taurine (WT, WCT, WTG) but also on the loading plots. The peak at 1033 cm^−1^ did not appear in the mixture sample, but there was a distinct peak at 1047 cm^−1^ in samples containing taurine. This peak was also observed in the loading plots. In the case of creatine, a peak at 828 cm^−1^ arising due to N–C = N scissoring [25] was observable in samples containing creatine. It can also be seen in the loading plots. The peak at 1394 cm^−1^ assigned to deformation and bending modes of CH_2_ and CH_3_ [25] had the same situation. The strong peak of glutamine was at 856 cm^−1^ [26], and it was more prominent in samples containing glutamine.

### 2.2. PCA Analysis

**Figure 2:**
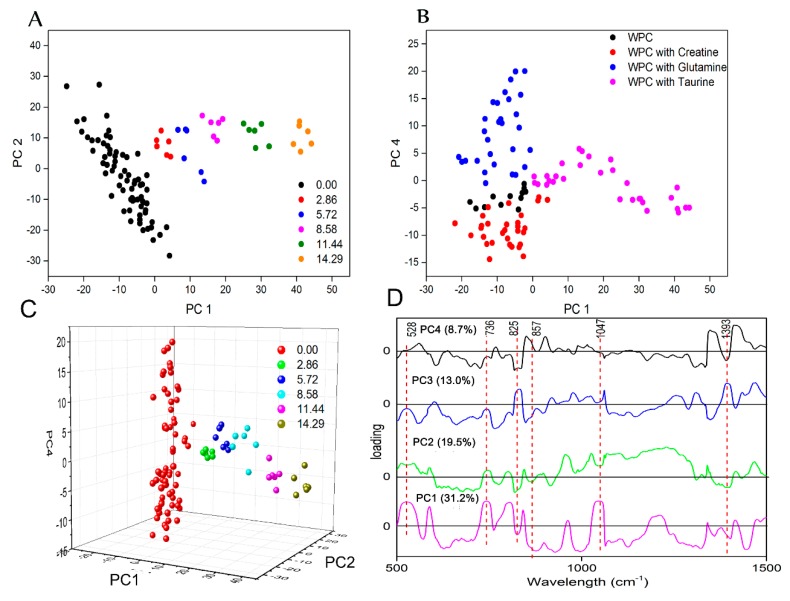
The results of principal component analysis (PCA) of samples (pure whey protein concentrate (WPC) and WPC mixed with one adulterant) in the 500–1500 cm^−1^ wavelength interval. (**A**) and (**C**) are the PCA scores colored by taurine concentration, (**B**) is the PCA scores of samples containing different adulterants, and (**D**) is the loading plots.

**Figure 3:**
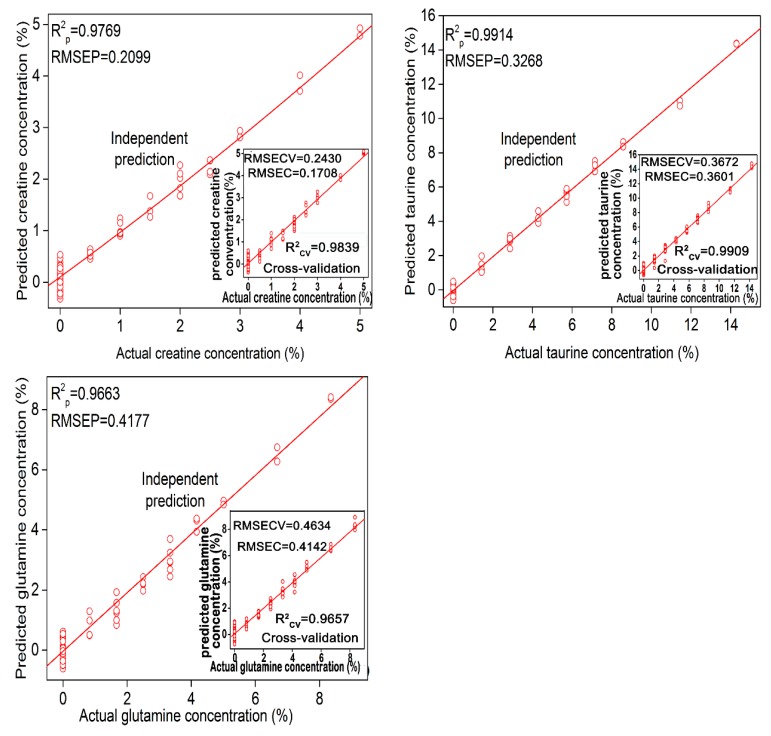
The partial least squares regression (PLSR) results of glutamine, creatine and taurine in the specific regions.

An anomaly spectrum was removed from the dataset by PCA. The PCA score plots were constructed using data in the range of 500–1500 cm^−1^ because it covers the major peaks of the adulterants. Figure 2A,B and Figure 3D show PCA scores plots on the PC1 (PC refers to principal component) vs. PC2 plane, PC1 vs. PC4 plane and PC1–PC2–PC4, respectively. The PCA loading plots can be seen in Figure 2C. A total of 73% of the total spectral variance was explained by the first four PCs. The first two PCs did not give a good separation of all classes but can completely separate samples adulterated with taurine from other samples based on the PC1 (Figure 2A,C). It can also be seen that samples with different taurine levels were clearly separated, and the score of PC1 enhanced with the increase of taurine concentration. PC1 and PC4 provided the best discrimination for four classes of samples (Figure 2B). It is clear that the sample containing taurine can be almost completely separated from the sample without taurine. The sample containing taurine was located on the right side of the figure and had a positive sign in the PC1, while the samples without taurine were opposite. Pure WPC, creatine-doped WPC and glutamine-doped WPC were separated by the PC4. The creatine-doped samples were partially separated from the pure WPC samples. Glutamine-doped WPC samples were located at the top left of the figure and were obviously separated from other samples. The loading plot shows that the important region for separating different adulterants is around 500–1100 cm^−1^, which contains the characteristic peaks of adulterants.

### 2.3. PLS-DA and SIMCA Classification Analysis

PLS-DA classification results are presented in Tables S2 and S4, and the calculated model efficiency parameters are displayed in Tables S3 and S5. Latent variables (LV) were selected for the PLS-DA model relied on section chemometrics analysis. The number of latent variables for the PLS-DA models, established with the full spectral range or 500–1100 cm^−1^, was 8. It is obvious that the PLS-DA classification results were general and the model constructed using the range of 500–1100 cm^−1^ with an accuracy of 82.8% was slightly better than that using the full spectrum range. The spectra that was not assigned to any classes in the training set and test set of the two models was zero. For the PLS-DA model based on the full spectrum, important information derived from the tables is that the pure WPC samples were correctly classified in both the training set and test set. The sensitivity and specificity of the W class were 100%, meaning that the model can completely distinguish pure WPC from adulterated samples. The ability of the model to correctly classify WC, WG and WT samples was also satisfactory, but the classification ability for WCG and WCT samples was weak, with a sensitivity in the training set of 65% and 50%, respectively. The model in the range of 500–1100 cm^−1^ can also perfectly separate pure WPC samples from adulterated samples. The sensitivity of the W class was 100% and the specificity was 98.3%. However, this model performed miserably for WCT and WTG classes. The sensitivity of WCT and WTG classes was 75% and 65% in training set.

The classification results and the calculated model efficiency parameters of the SIMCA model are listed in Table 1 and Table 2 and Tables S6 and S7. The results show that the SIMCA model with a range of 500–1100 cm^−1^ was satisfactory and superior to any other classification models. For the SIMCA model established based on the range of 500–1100 cm^−1^, the principal components of classes were 2, 5, 4, 5, 5, 4, 4, respectively. The cumulative explained variance of each class using chosen the principal components was approximately 92%. The sensitivity of all classes was greater than 90%, except for the W class in the test samples. The accuracy of the entire calibration model was more than 97.6% and the error rate was less than 2.1%, indicating that the developed model has great potential to discriminate different adulterated samples with 95% confidence levels. The results from the test samples were equally surprising. Only two samples were misclassified and the error rate was 5%, which further confirmed this conclusion. The results of the training set and test set were similar, which indicates that the established model is robust. The spectral information of adulterants is mainly in the range of 500–1100 cm^−1^, and the other ranges are mainly Raman information of whey protein. Since we mainly classify samples with different kinds of adulterants, the use of the range of 500–1100 cm^−1^ for classification prediction can effectively avoid the influence of other intervals spectra and noise. Therefore, the performance of the model based on 500–1100 cm^−1^ was better than that using the full spectral range.

### 2.4. PLSR Analysis

Table 3 lists the parameters and results of the best PLSR models for the quantitative determination of different adulterants. The limit of detection and quantification values for all adulterants are also displayed in Table 3. The root mean squared error on the cross-validation (RMSECV) values were highly similar to the root mean squared error of prediction (RMSEP) values, indicating that there is no overfitting of the models. The determination coefficients of all models were greater than 0.96. The concentration of taurine that can be detected using the full spectral range was 0.83%, whereas the lowest detectable concentration was 0.71% in the optimal region of 500–1100 cm^−1^. The limit of quantification for taurine in the optimal range (2.15%) was lower than that in the full spectral range (2.52%). Using a specific spectral range to predict the content of taurine was the best choice and it obtained a determination coefficient of 0.991 and a root mean square error of 0.33%. The optimal range for glutamine was the combination of range 800–1000 cm^−1^ and 1300–1500 cm^−1^. The best predictive precision, detection and quantification results were in the optimal range. RMSEP, limit of detection (LOD) and limit of quantification (LOQ) values for glutamine in the optimal range were 0.42%, 1.13% and 3.40%, respectively. In the case of creatine, the optimal range was also 500–1100 cm^−1^ with LOD, LOQ and RMSEP values of 0.50%, 1.52% and 0.22%, respectively. The performance of the model based on the full spectrum (RMSEP = 0.23%, LOD = 0.66% and LOQ = 2.00%) was slightly inferior. In view of the above, the prediction models of various adulterants built based on the characteristic spectral range were superior to the models established using the full spectral range. The characteristic spectral range of the component contains a large amount of spectral information of adulterants and is most sensitive to the changes of adulterant content. However, other intervals do not contain its characteristic variables, and band redundancy exists for a certain adulterant. Using the characteristic spectral range of the component for prediction can effectively avoid the influence of other intervals’ spectra and noise. Therefore, the prediction result using a characteristic spectral range of the component is generally better than using the whole region. Figure 3 displays the best PLSR prediction model for each adulterant.

## 3. Material and Methods

### 3.1. Preparation of the Samples

WPC with a declared protein content of 80% by the supplier was provided by Yinuo Biotechnology Co. Ltd. (Hangzhou, China). l-glutamine, creatine and taurine were purchased from Aladdin Reagent Company (Shanghai, China).

A total of 32 samples (30 adulterated, 2 pure WPC) were prepared according to the Matyas Lukacs method [27] and two copies of each sample were prepared. All substances were powder with uniform particles except for taurine, which was ground before being added to the WPC. A total of 10 g of mixture powder for each sample was prepared. The corresponding quantity of the adulterants was added to the WPC and shaken violently in a plastic bottle for 5 min to make it evenly mixed. Table S1 displays the adulterated substances used and their content changes in the samples. Before the Raman test for each sample, 1 g (accurate to 0.0001 g) sample was dissolved in 5 mL deionized water and placed in the ultrasonic machine for 4 min to make it fully dissolved. The samples in this experiment were prepared by the dry-blended method and the particle size of the whey protein powder and adulterants was larger than the size of confocal Raman laser spot (about 3 μm). Solid l-glutamine, creatine and taurine were directly measured.

### 3.2. Raman Spectroscopy

A Renishaw InVia confocal Raman spectrometer (Renishaw Plc. Gloucestershire, UK), equipped with a charge-coupled device (CCD) camera operated at −75 °C, and a semiconductor laser emitting a wavelength of 785 nm was employed to acquire the Raman spectra. Biological samples emit fluorescence by themselves under the irradiation of light. Laser excitation of these samples by 488 nm or 532 nm will produce strong fluorescence signals. The intensity of these fluorescence signals is much stronger than that of Raman signals, which will mask the Raman signals. The use of a 785-nm laser reduces the interference of fluorescence on the Raman signal, so we chose to use a laser with an excitation wavelength of 785 nm in this experiment. Exposure time, accumulation time and laser power were explored in the preliminary Raman measurements to obtain good signal noise ratio and minimal fluorescence interference. The choice of these parameters also ensures that the sample is not knocked and damaged during the experiment. The optimized conditions of the test were 250 scans, 0.2 s exposure time and 140 mW laser power. Spectra were obtained in the fingerprint region from 400 to 1800 cm^−1^ with a spectral resolution of approximately 1 cm^−1^, in which 20× objective was applied to focus the laser on samples. The Raman system was calibrated using silicon wafers prior to the experiment to reduce the variation caused by instrument components.

The dissolved sample was directly dropped on a glass slide with a 3-mm deep groove and immediately placed on the Raman device for spectral scanning. Each sample was measured at three different positions, leading to a total of 192 spectra. All Raman experiments were carried out in a dark room at 26 °C to eliminate the interference of natural light on the experiment.

To observe the characteristic absorption peaks of the sample, the obtained spectrum which contains fluorescent noise needs to be pretreated. All spectra were first manually removed by cosmic rays, and then subjected to a baseline correction using a first-order polynomial fitting. Subsequently, Sawiztky–Golay (SG) filtering was utilized to smooth the spectrum, and the parameters were second-order polynomial with 25 smoothing points.

### 3.3. Chemometric Analysis

In this study, the outliers were discovered and deleted with the aid of robust PCA [28]. The PCA model was built using additional normalized data in the range of 500–1500 cm^−1^ for 102 Raman spectra (pure WPC, WPC adulterated with creatine, WPC adulterated with glutamine, WPC adulterated with taurine) to observe whether the samples have natural grouping.

For classifying different adulterated samples, the multivariate classification methods PLS-DA and SIMCA were used. In order to obtain the best prediction model, the full spectral range and 500–1100 cm^−1^ range [29] were respectively used to construct the classification model. The validation procedure adopted k-fold cross-validation where K is equal to 7. The latent variables with the smallest cross-validation error in classification were selected for PLS-DA. A smaller number of potential variables is preferred to avoid model overfitting when RMSECV values are not significantly different. The number of principal components with less error rate in the classification were chosen for SIMCA [30]. All data were split into two sets called the training set and test set by the Kennard–Stone (KS) algorithm. A total of 66% of the data was assigned to the training set and 34% of the data was divided into the test set.

For evaluating the classification performance of the developed model, sensitivity, specificity (also known as selectivity), precision, Younden’s index, accuracy, error rate and no-error rate were calculated [30,31,32,33]. Sensitivity represents the ability that the method correctly identifies a positive sample as positive. Specificity refers to the probability of correct classification of negative samples. Precision (also called false positive rate) is the probability that a negative sample is wrongly identified as a positive sample by the model. The ratio of all correctly identified samples to total samples represents accuracy. The global performance of different classification methods was compared using the area under the curve (AUC), where the value is 1 when model has the strongest classification ability. These parameters were calculated as follows:

$$\begin{array}{l} Sensitivity=\frac{TP}{TP+FN}\\ Specificity=\frac{TN}{FP+TN}\\ precision=\frac{TP}{TP+FP}\\ younden’s\;index=Sensitivity+Selectivity-1\\ Accuracy=\frac{TP+TN}{TP+FN+FP+TN}\;\\ Non-error\;rate=\frac{\sum_{1}^{n}Seneitivity}{n}\\ Error\;rate=1-non\;error\;rate \end{array}$$

where (true positive) TP, true negative (TN), false positive (FP) and false negative (FN) represent the number of samples observed.

Partial least squares regression (PLSR) is a multivariate analysis method diffusely applied with Raman spectroscopy. It was used to predict the concentration of adulterants in this paper, and the calibration model which was built based on the Raman spectrum and actual values using a 7-fold cross-validation mode to test. The number of latent variables was determined by the minimum root mean squared error on the cross-validation set (RMSECV) [22]. The determination coefficients (R^2^) and root mean square errors (RMSE) of both training and test data sets were applied to evaluate the performance of the PLSR model. The model has the best performance when the RMSE value is the smallest and the R^2^ value is close to 1. The robustness of the models was tested by test set data that was not involved in the model establishment. R^2^, RMSE values were calculated as follows:

$$\begin{array}{l} \mathrm{RMSE}=\sqrt{\frac{\sum_{i=1}^{n}{\left({\hat{y}}_{i}-y_{i}\right)}^{2}}{n}}\\ R^{2}=1-\frac{\sum_{i=1}^{n}{\left(y_{i}-{\hat{y}}_{i}\right)}^{2}}{\sum_{i=1}^{n}{\left(y_{i}-\bar{y}\right)}^{2}} \end{array}$$

Here, n represents the number of samples in the training set or test set, $y_{i}$ is the reference value, ${\hat{y}}_{i}$ is the predicted value by the PLSR model and $\bar{y}$ equals the average value of all reference values.

The dataset of 191 spectra was also divided into training set (127 spectra) and test set (64 spectra) as section PLS-DA. The training set was used for calibration and the test set was used to test the predictive ability of the model. In order to obtain better prediction models for different adulterants, the full spectrum (400–1800 cm^−1^) and the characteristic spectral range of different adulterants were used to predict the adulteration concentration. Additional data pretreatment methods including standard normal variation (SNV), normalization, first derivative, second derivative and multiple scattering calibration (MSC) were selected as the most proper for different adulterants. The limit of detection (LOD) and limit of quantification (LOQ), that represent the minimum detectable concentration or quantitative concentration based on the established PLSR models, were calculated according to the method described by Banu Sezer, using the following equations [34]:

$$LOD=3.3\;\frac{SD}{S}$$

$$LOQ=10\;\frac{SD}{S}$$

where SD represents the standard deviation of the predicted concentration for unadulterated samples and S is the slop of the calibration curve.

Multivariate data analysis PCA, PLSR, PLS-DA and SIMCA were performed in SIMCA-P14.1.

## 4. Conclusions

This paper proved that Raman spectroscopy combined with multivariate analysis can quickly detect three common adulterants (creatine, taurine and glutamine) in whey protein powder. The PCA analysis highlight that pure WPC samples and three types of samples containing only one adulterant can be clearly separated from each other. Raman spectroscopy was applied jointly with PLS-DA and SIMCA to discriminate the samples with different adulterants. The SIMCA model, which was built based on 500–1100 cm^−1^, was the most effective model with an accuracy of 96.9% and an error rate of 5%. The combination of Raman spectroscopy and PLSR analysis can sensitively detect the adulterate content, and different pretreatment methods and spectral intervals were used to model for each adulterant content. The PLSR model based on typical Raman regions (500–1100 cm^−1^ for creatine and taurine, the combination of range 800–1000 cm^−1^ and 1300–1500 cm^−1^ for glutamine) for adulterants had high R^2^ and low RMSEP values, and had better performance than that based on the full spectral region. RMSEP was 0.21%, 0.33%, 0.42% for creatine, taurine and glutamine. The limit of detection was below 1.13%. This method is fast, simple and accurate compared with other methods. The sample only needs simple dissolution pretreatment and is very suitable for adulteration analysis of WPC in industry or daily life.

## Figures and Tables

**Table 1:** Classification prediction results and number of components of soft independent modeling class analogy (SIMCA) model with 500–1100 cm^−1^ spectral regions.

	Number of Components	Members	Correct	W	WC	WG	WT	WCG	WCT	WTG	No Class
Training Set											
**W**	2	7	100%	7	0	0	0	0	0	0	0
**WC**	5	20	100%	0	20	0	0	0	0	0	0
**WG**	4	20	100%	0	0	20	0	0	0	0	0
**WT**	5	20	100%	0	0	0	20	0	0	0	0
**WCG**	5	20	95%	0	0	0	0	19	1	0	0
**WCT**	4	20	100%	0	0	0	0	0	20	0	0
**WTG**	4	20	90%	0	0	0	2	0	0	18	0
**Total**		127	97.6%	7	20	20	22	19	21	18	0
**Test set**											
**W**		4	75%	3	1	0	0	0	0	0	0
**WC**		10	100%	0	10	0	0	0	0	0	0
**WG**		10	90%	1	0	9	0	0	0	0	0
**WT**		10	100%	0	0	0	10	0	0	0	0
**WCG**		10	100%	0	0	0	0	10	0	0	0
**WCT**		10	100%	0	0	0	0	0	10	0	0
**WTG**		10	100%	0	0	0	0	0	0	10	0
**Total**		64	96.9%	4	11	9	10	10	10	10	0

**Table 2:** The classification performance parameters of the SIMCA model with 500–1100 cm^−1^ spectral regions. The first value is the training group, and the latter value represents the test group.

	W	WC	WG	WT	WCG	WCT	WTG
**Sensitivity**	100%/75%	100%/100%	100%/90.0%	100%/100%	95%/100%	100%/100%	90%/100%
**Specificity**	100%/98.3%	100%/98.1%	100%/100%	98.1%/100%	100%/100%	99.1%/100%	100%/100%
**Precision**	100%/75%	100%/90.9%	100%/100%	90.9%/100%	100%/100%	95.2%/100%	100%/100%
**Accuracy**	97.6%/96.9%						
**No-error rate**	97.9%/95.0%						
**Error rate**	2.1%/5.0%						
**Younden’s index**	100%/73.3%	100%/98.1%	100%/90.0%	98.1%/100%	95%/100%	99.1%/100%	90%/100%
**AUC (training set)**	1.00	0.99	1.00	0.99	1.00	0.98	0.98

**Table 3:** Partial least squares regression (PLSR) results for three adulterate concentrations in different spectral regions. ^a^ is the pretreatment method used; ^b^ refers to determination coefficient of cross-validation; ^c^ is the determination coefficient of prediction; ^d^ represents the room mean square error of calibration; ^e^ is the room mean square error of cross-validation; ^f^ is the room mean square error of prediction; ^g^ and ^h^ are limit of detection and limit of quantification; ^j^ is the number of latent variables.

**Entire Region**	**400–1800 cm^−1^**		
	**Taurine**	**Glutamine**	**Creatine**
Pre-treat ^a^	SG + SNV	SG + SNV	SG + 1st der.
LV ^j^	2	4	4
R^2^cv ^b^	0.991	0.970	0.972
R^2^p ^c^	0.992	0.963	0.967
RMSEC ^d^ (%)	0.37	0.39	0.22
RMSECV ^e^ (%)	0.41	0.48	0.25
RMSEP ^f^ (%)	0.35	0.43	0.23
LOD ^g^ (%)	0.83	1.17	0.66
LOQ ^h^ (%)	2.52	3.51	2.00
**Component-specific range**	**500–1100 cm^−1^**	**800–1000 cm^−1^ and** **1300–1500 cm^−1^**	**500–1100 cm^−1^**
Pre-treat ^a^	SG + MSC	SG	SG + MSC
LV ^j^	6	8	6
R^2^cv ^b^	0.991	0.966	0.984
R^2^p ^c^	0.991	0.966	0.977
RMSEC ^d^ (%)	0.36	0.41	0.17
RMSECV ^e^ (%)	0.37	0.46	0.24
RMSEP ^f^ (%)	0.33	0.42	0.21
LOD ^g^ (%)	0.71	1.13	0.53
LOQ ^h^ (%)	2.15	3.40	1.52

## Supplementary Materials

The supplementary materials are available online.
