# Supplementary material for: Rapid Detection of Adulterants in Whey Protein Supplement by Raman Spectroscopy Combined with Multivariate Analysis
Source: Molecules. 2019 May 16;24(10):1889. doi: 10.3390/molecules24101889 (PMC6571825; doi:10.3390/molecules24101889)
Supplement: Supplementary file 1 [file molecules-24-01889-s001.pdf]

Table S1. Composition of 32 WPC samples used in the experiment.

Sample number	Creatine (%)	Glutamine (%)	Taurine (%)	WPC (%)
1	1.00	0.00	0.00	99.00
2	2.00	0.00	0.00	98.00
3	3.00	0.00	0.00	97.00
4	4.00	0.00	0.00	96.00
5	5.00	0.00	0.00	95.00
6	0.00	1.67	0.00	98.33
7	0.00	3.34	0.00	96.66
8	0.00	5.01	0.00	94.99
9	0.00	6.68	0.00	93.32
10	0.00	8.35	0.00	91.65
11	0.00	0.00	2.86	97.14
12	0.00	0.00	5.72	94.28
13	0.00	0.00	8.58	91.42
14	0.00	0.00	11.44	88.56
15	0.00	0.00	14.29	85.71
16	0.50	0.83	0.00	98.67
17	1.00	1.67	0.00	97.33
18	1.50	2.50	0.00	96.00
19	2.00	3.34	0.00	94.66
20	2.50	4.17	0.00	93.33
21	0.50	0.00	1.43	98.07
22	1.00	0.00	2.86	96.14
23	1.50	0.00	4.29	94.21
24	2.00	0.00	5.72	92.28
25	2.50	0.00	7.15	90.35
26	0.00	0.83	1.43	97.74
27	0.00	1.67	2.86	95.47
28	0.00	2.50	4.29	93.21
29	0.00	3.34	5.72	90.94
30	0.00	4.17	7.15	88.68
31	0.00	0.00	0.00	100
32	0.00	0.00	0.00	100

Table S2. Classification predictions results and number of components of Partial least squares discriminant analysis (PLS-DA) model with full-spectra.

	Members	Correct	W	WC	WG	WT	WCG	WCT	WTG	No class
**Number of latent variables 8**										
**Training set**										
**W**	7	100%	7	0	0	0	0	0	0	0
**WC**	20	95%	0	19	0	0	1	0	0	0
**WG**	20	85%	0	0	17	0	3	0	0	0
**WT**	20	95%	0	0	0	19	0	1	0	0
**WCG**	20	65%	0	1	4	0	13	1	1	0
**WCT**	20	50%	0	2	0	3	2	10	3	0
**WTG**	20	75%	0	0	0	4	0	1	15	0
**Total**	127	78.7%	7	22	21	26	19	13	19	0
**Test set**										
**W**	4	100%	4	0	0	0	0	0	0	0
**WC**	10	100%	0	10	0	0	0	0	0	0
**WG**	10	90%	0	0	9	0	1	0	0	0
**WT**	10	90%	0	0	0	9	0	0	1	0
**WCG**	10	90%	0	0	0	0	9	0	1	0
**WCT**	10	40%	0	3	0	2	1	4	0	0
**WTG**	10	70%	0	0	0	2	1	0	7	0
**Total**	64	81.3%	4	13	9	13	12	4	9	0

Table S3. The classification performance parameters of PLS-DA model with full-spectra. The values before the diagonal line belong to the training set, and the values after the diagonal line are for the test set. This also applies to Table S4-S7.

	W	WC	WG	WT	WCG	WCT	WTG
**Sensitivity**	100%/100%	95%/100%	85%/90%	95%/90%	65%/90%	50%/40%	75%/70%
**Specificity**	100%/100%	97.2%/94.4%	96.3%/100%	93.5%/92.6%	94.4%/94.4%	97.2%/100%	96.3%/96.3%
**Precision**	100%/100%	86.4%/76.9%	81.0%/100%	73.1%/69.2%	68.4%/75.0%	76.9%/100%	78.9%/77.8%
**Accuracy**	78.7%/81.3%						
**NO-error rate**	80.7%/82.3%						
**Error rate**	19.3%/17.7%						
**Younden's index**	100%/100%	92.2%/94.4%	81.3%/90%	88.5%/82.6%	59.4%/84.4%	47.2%/40%	71.3%/66.3%
**AUC（training set）**	1.00	0.99	0.96	0.97	0.96	0.86	0.92

Table S4. Classification predictions results and number of components of PLS-DA model with 500-1100 cm^{-1} spectral regions.

	Members	Correct	W	WC	WG	WT	WCG	WCT	WTG	No class
**Number of latent variables**	8									
**Training set**										
**W**	7	100%	7	0	0	0	0	0	0	0
**WC**	20	100%	0	20	0	0	0	0	0	0
**WG**	20	80%	0	0	16	0	2	0	2	0
**WT**	20	95%	0	0	0	19	0	1	0	0
**WCG**	20	80%	0	1	2	0	16	0	1	0
**WCT**	20	75%	0	1	1	2	0	15	1	0
**WTG**	20	65%	0	0	2	1	2	2	13	0
**Total**	127	83.5%	7	22	21	22	20	18	17	0
**Test set**										
**W**	4	100%	4	0	0	0	0	0	0	0
**WC**	10	100%	0	10	0	0	0	0	0	0
**WG**	10	80%	1	0	8	0	0	0	1	0
**WT**	10	90%	0	0	0	9	1	0	0	0
**WCG**	10	100%	0	0	0	0	10	0	0	0
**WCT**	10	60%	0	2	0	1	1	6	0	0
**WTG**	10	60%	0	0	2	1	1	0	6	0
**Total**	64	82.8%	5	12	10	11	13	6	7	0

Table S5. The classification performance parameters of PLS-DA model with 500-1100 cm^{-1} spectral regions.

	W	WC	WG	WT	WCG	WCT	WTG
**Sensitivity**	100%/100%	100%/100%	80%/80%	95%/90%	80%/100%	75%/60%	65%/60%
**Specificity**	100%/98.3%	98.1%/96.3%	95.3%/96.3%	97.2%/96.3%	96.3%/94.4%	97.2%/100%	96.3%/98.1%
**Precision**	100%/80.0%	90.9%/83.3%	76.2%/80.0%	86.4%/81.8%	80.0%/76.9%	83.3%/100%	76.5%/85.7%
**Accuracy**	83.5%/82.8%						
**No-error rate**	85.0%/84.3%						
**Error rate**	15.0%%/15.7%						
**Younden's index**	100%/98.3%	98.1%/96.3%	75.3%/76.3%	92.2%/86.3%	76.3%/94.4%	72.2%/60%	61.3%/58.1%
**AUC（training set）**	1.00	0.99	0.98	0.98	0.98	0.87	0.89

Table S6. Classification predictions results and number of components of Soft independent modelling class analogy (SIMCA) model with full-spectra.

	Number of components	Members	Correct	W	WC	WG	WT	WCG	WCT	WTG	No class
**Training set**											
**W**	1	7	85.7%	6	1	0	0	0	0	0	0
**WC**	5	20	60%	8	12	0	0	0	0	0	0
**WG**	5	20	80%	4	0	16	0	0	0	0	0
**WT**	6	20	95%	0	0	0	19	0	1	0	0
**WCG**	7	20	90%	2	0	0	0	18	0	0	0
**WCT**	4	20	100%	0	0	0	0	0	20	0	0
**WTG**	6	20	75%	3	0	0	0	0	2	15	0
**Total**		127	83.5%	23	13	16	19	18	23	15	0
**Test set**											
**W**		4	100%	4	0	0	0	0	0	0	0
**WC**		10	50%	4	5	0	0	0	1	0	0
**WG**		10	70%	2	0	7	0	1	0	0	0
**WT**		10	90%	0	0	0	9	0	1	0	0
**WCG**		10	80%	1	0	0	1	8	0	0	0
**WCT**		10	80%	2	0	0	0	0	8	0	0
**WTG**		10	70%	1	0	0	0	0	2	7	0
**Total**		64	75.0%	14	5	7	10	9	12	7	0

Table S7. The classification performance parameters of SIMCA model with full-spectra.

	W	WC	WG	WT	WCG	WCT	WTG
**Sensitivity**	85.7%/100%	60%/50%	80%/70%	95%/90%	90%/80%	100%/80%	75%/70%
**Specificity**	85.8%/83.3%	99.1%/100%	100%/100%	100%/98.1%	100%/98.1%	97.2%/92.6%	100%/100%
**Precision**	26.1%/28.6%	92.3%/100%	100%/100%	100%/90.0%	100%/88.9%	87.0%/66.7%	100%/100%
**Accuracy**	83.5%/75.0%						
**No-error rate**	83.7%/77.1%						
**Error rate**	16.3%/22.9%						
**Younden's index**	71.5%/83.3%	59.1%/50%	80%/70%	95%/88.1%	90%/78.1%	97.2%/72.60%	75%/70%
**AUC（training set）**	0.91	0.99	1.00	0.99	1.00	0.95	1.00
